# Home-Based, Remotely Supervised Transcranial Direct Current Stimulation Improves the Overall Pain Experience of Older Adults With Knee Osteoarthritis

**DOI:** 10.1155/prm/1783171

**Published:** 2025-02-24

**Authors:** Chiyoung Lee, Juyoung Park, C. Kent Kwoh, Mindy Fain, Lindsey Park, Hyochol Ahn

**Affiliations:** ^1^The University of Arizona College of Nursing, Tucson, Arizona, USA; ^2^The University of Arizona Arthritis Center, Tucson, Arizona, USA; ^3^Division of Rheumatology, The University of Arizona College of Medicine, Tucson, Arizona, USA; ^4^Division of General Internal Medicine, Geriatrics and Palliative Medicine, The University of Arizona College of Medicine, Tucson, Arizona, USA; ^5^The University of Arizona Center of Aging, Tucson, Arizona, USA

**Keywords:** knee osteoarthritis, latent transition analysis, older adults, pain, transcranial direct current stimulation

## Abstract

**Objective:** Chronic pain in knee osteoarthritis (OA) is a multidimensional phenomenon requiring thorough assessment and appropriate treatment. We assessed the impact of home-based, remotely supervised transcranial direct current stimulation (tDCS) on the overall pain experience of older adults with knee OA by simultaneously examining its effects on multiple pain domains—pain intensity, pain interference, and pain catastrophizing—using multigroup latent transition analysis (LTA).

**Methods:** This secondary analysis of a randomized clinical trial involved 120 participants with knee OA pain, randomly assigned in a 1:1 ratio to receive 15 daily sessions of 2-mA tDCS or sham tDCS (20 min per session) over three weeks, with real-time remote supervision. Pain intensity was measured using the Numeric Rating Scale (NRS) and the pain subscale of the Western Ontario and McMaster Universities Osteoarthritis (WOMAC) Index. Pain interference was measured using the WOMAC functional scale. Pain catastrophizing was assessed using the Pain Catastrophizing Scale (PCS). All the measures were assessed at baseline and at the end of each week (weeks 1, 2, and 3), after the participants had completed five tDCS sessions per week. Multigroup LTA enabled the simultaneous measurement of multiple pain domains and analysis of their changes as a function of intervention exposure by modeling the transition probabilities of latent classes and comparing these changes between the groups.

**Results:** Based on the NRS, WOMAC, and PCS scores, three latent categories were identified: “high pain (all scores high),” “moderate pain (all scores moderate),” and “low pain (all scores low).” Active group participants with “moderate pain” at baseline had a 24.2% probability of transitioning to “low pain” after Week 1, whereas sham group participants remained stagnant during this interval. Notably, 37.6% of active group participants with “high pain” at Week 1 transitioned to “moderate pain,” while 35.8% of those with “moderate pain” at Week 1 transitioned to “low pain” by Week 2 (after an additional five sessions). Nevertheless, no noticeable changes were observed in the sham group during this period. No pronounced intervention effects were noted by Week 3.

**Conclusions:** Simultaneously modeling pain-related measures enriches our understanding of the efficacy of tDCS in improving the overall pain experience among older adults with knee OA.

**Trial Registration:** ClinicalTrials.gov identifier: NCT04016272

## 1. Introduction

Osteoarthritis (OA) is a commonly reported joint disease in older adults, with knee OA being particularly prevalent [1]. Although knee OA is characterized by numerous hallmarks, pain is the predominant symptom in older adults that leads to functional limitations, physical disability, and an overall reduction in quality of life (QoL) [1–3]. Consequently, pain is the primary treatment target for this condition.

The management of knee OA pain is challenging, especially in older adults. Pharmacological treatments often provide limited relief and carry the risk of adverse side effects [4]. Surgical options may not always be feasible owing to preexisting comorbidities. Considering these limitations, other nonpharmacologic approaches have been evaluated. In particular, several researchers have investigated the effects of interventions targeting the central nervous system, such as transcranial direct current stimulation (tDCS), which has demonstrated promise in alleviating knee OA pain [5, 6].

Briefly, tDCS is a noninvasive method that modulates cortical excitability by applying low-amplitude direct currents to the scalp. Electrical stimulation of the primary motor cortex can modulate pain by activating thalamic nuclei directly connected to the motor and premotor cortices and stimulating the medial thalamus, anterior cingulate, and upper brainstem [7, 8]. Increasing research into tDCS has revealed its potential in managing knee OA pain, not only in clinical settings but also via home-based, remotely supervised applications, thus enhancing its accessibility and convenience for those with limited mobility, especially older adults [9].

To date, assessments of tDCS's impact on pain have primarily used conventional metrics for clinical pain intensity [10, 11]. However, chronic OA pain is a multidimensional, composite experience that requires thorough assessment and is not limited to pain intensity alone [12]. Pain interference, which is the perceived pain-induced disruption of daily living, is also a critical aspect of the overall pain experience of older adults with knee OA, strongly influencing their QoL [13]. Furthermore, pain catastrophizing, the tendency to have an exaggerated negative view of pain, affects overall pain by modulating supraspinal pain processing, attentional bias, and altered central nervous system processes and directly contributes to analgesic use, increased pain, and illness-related behaviors [14]—all of which necessitate inclusion as vital parameters in chronic pain evaluation and treatment.

Importantly, employing an approach that *simultaneously* leverages these measures may enhance the reliability of pain evaluations and facilitate a more comprehensive assessment of tDCS's efficacy in improving the overall pain experience among older adults with knee OA. Assessing the intervention's impact on different pain domains separately might reveal improvements in specific areas, as seen in other studies [10, 11]. However, because these pain domains are interrelated and interactive [15], evaluating them together offers a more realistic view of the intervention's efficacy. This integrated approach also better reflects the complex nature of chronic pain and provides fresh insights into the multifaceted impact of tDCS, which constitutes our primary aim.

We endeavored to achieve our aim using a unified analysis approach called latent transition analysis (LTA). LTA is a person-centered approach that uses longitudinal data to handle transitions across latent classes of individuals over time (e.g., movements between different pain profile groups) [16]. In LTA, these latent classes are considered dynamic “statuses” rather than stable classifications that people may move in and out of over time; such movement between latent statuses is quantified in a matrix of *transition probabilities* [16]. Therefore, LTA enables researchers to estimate the transition probabilities between different pain profiles (e.g., from a high pain status to a moderate pain status). In clinical trials, the use of multigroup LTA with the grouping variable as the intervention condition can help estimate the group-specific probability of belonging to a latent status at each time point as well as the group-specific transition probability over time, thus potentially modulating interventional efforts that promote symptom improvement [17, 18]. Most importantly, compared to traditional approaches, such as growth curve modeling and repeated-measures analysis of variance, another strength of LTA is that it studies multiple indicators (i.e., evaluates the contributions of different pain-related measures to each latent status) [16], thereby elucidating the *overall pain experience* and *their simultaneous changes* as a function of intervention exposure. Thus, LTA captures the multidimensional nature of chronic pain experiences rather than treating them as unidimensional.

### 1.1. Aim of the Study

This study aimed to evaluate the efficacy of tDCS in improving the overall pain experience in older adults with knee OA by simultaneously examining its effects on multiple pain domains—pain intensity, pain interference, and pain catastrophizing—using the multigroup LTA framework. Our trial innovatively comprised a 3-week home-based tDCS program monitored in real time via secure videoconferencing. Changes in pain outcomes were evaluated from baseline through 3 weeks, with assessments at the end of each week following five sessions per week. Ultimately, this design aimed to provide insights into the weekly progression of pain management in knee OA. We hypothesize that tDCS will cause simultaneous improvements in multiple pain indicators, with progressive benefits observed every week throughout the intervention process.

## 2. Methods

### 2.1. Design

**Figure 1:**
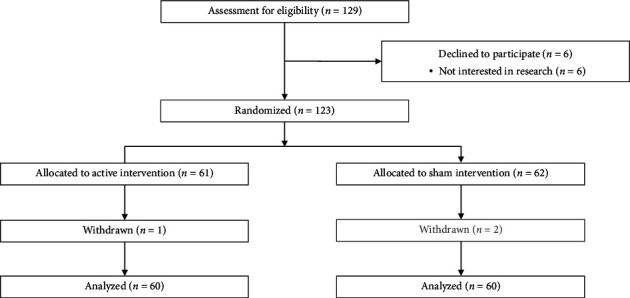
Participant flow diagram (*N* = 120). *Note:* “Declined” meant that participants came to baseline visit (signed informed consent) but did not start any intervention. For this study, among 6 declined subjects, 4 subjects signed informed consent at the baseline but declined to study participation; 2 subjects did not show up at the baseline.

This is a secondary analysis of a double-blind, randomized, sham-controlled, phase II, parallel-group pilot clinical trial. A total of 120 eligible participants, who provided informed and written consent, were randomly assigned to one of the two groups: active tDCS and sham tDCS, with each group comprising 60 participants (*n* = 60) (Figure 1). Participant assignment relied on the order of study entries and a pregenerated randomization list, which was created via SAS software (Version 9.4) by a statistician uninvolved in the trial's clinical aspects. To achieve a covariate balance between the groups, covariate adaptive randomization was employed.

### 2.2. Participants

Eligible participants were individuals aged 50–85 who (1) had symptomatic knee OA diagnosed according to the American College of Rheumatology criteria (knee radiographs were utilized to determine the severity of OA using Kellgren–Lawrence scores); (2) experienced knee OA pain in the past three months, with an average pain score of at least 30 on a 0–100 Numeric Rating Scale (NRS); (3) could speak and read English; and (4) had no plans to change pain medication regimens during the trial. According to the American College of Rheumatology criteria for classifying OA, participants were required to meet at least three of six criteria, which included age > 50 years, stiffness lasting < 30 min, presence of crepitus, bony tenderness, bony enlargement, and absence of palpable warmth.

Exclusion criteria encompassed medical conditions that could potentially impact result interpretation, pose safety concerns during assessments or tDCS procedures, or hinder the successful completion of the study protocol. Specific exclusions comprised individuals who (1) had undergone prosthetic knee replacement or nonarthroscopic surgery on the affected knee; (2) had a history of brain surgery, brain tumor, seizure, stroke, or intracranial metal implants; (3) were diagnosed with systemic rheumatic diseases such as rheumatoid arthritis, systemic lupus erythematosus, or fibromyalgia; (4) exhibited alcohol/substance abuse; (5) were currently undergoing treatment with sodium channel blockers, calcium channel blockers, or NMDA receptor antagonists; (6) demonstrated reduced cognitive abilities (i.e., a Mini-Mental Status Exam score ≤ 23) that could hinder comprehension of the study procedures; (7) were pregnant or breastfeeding; (8) had experienced psychiatric hospitalization in the past year; and (9) lacked access to the internet.

Participants were recruited from Southeast Texas by advertising the study at local institutions such as UTHealth and in nearby communities through flyers. Additionally, potential participants were directly identified and recruited from the UT Physicians Orthopedics Clinic.

### 2.3. Intervention

Active tDCS. The tDCS device was a “Soterix 1 × 1 tDCS mini-CT Stimulator” (Soterix Medical Inc., NY) equipped with headgear and 5 × 7 cm saline-soaked surface sponge electrodes. The sponge electrodes were fixed to the custom headgear, which was easily secured onto the participant's head to ensure a simple and foolproof electrode setup [19]. This single-position headgear, clearly marked with labeled sponges, mitigates user error and ensures accurate montage placement. The anode was placed over the primary motor cortex (M1) and the cathode over the contralateral supraorbital area. Anodal stimulation over M1 activates various afferent and efferent neural circuits, leading to improvements in pain and psychological symptoms [20, 21]. A constant current of 2 mA (subthreshold intensity) was applied for 20 min, with 30 s of ramp-up and 30 s of ramp-down to ensure reliable blinding [20], for each session, totaling 15 sessions over 3 weeks (five sessions per week).

Participants received comprehensive training on the usage of the tDCS device during their baseline visit; they were shown how to apply and operate the device, practiced under supervision, and received feedback until they were comfortable using it independently. Once the research staff had verified that the participant had understood all the details of the stimulation procedure, they provided the participant with a tDCS device and a daily organized device kit, accompanied by a pictorial manual with written instructions. Participants could only undergo a stimulation session strictly after having received a unique unlock code from the research team. Once suitable contact quality had been achieved, they could then proceed to the next step, which is to initiate stimulation, albeit without altering the device's settings. This device features double-blinding protocols that necessitate entering a five-digit code to initiate stimulation. Each unique code, intended to trigger the programmed stimulation sequence, is for one-time use and can only be activated once. Both the experimenter and participant were unaware of the group assignments. After entering the unlock code, the device displayed a countdown timer for the 20-min session. Once the time had elapsed, the device automatically switched off, and participants were instructed to remove and dispose of the sponges and securely store the equipment for the subsequent session.

Sham tDCS. For sham stimulation, the setup mirrored that of the active one. However, the stimulator was only activated for 30 s at the beginning and end of the session to replicate the sensory experience of active tDCS without delivering a sustained current, effectively concealing whether the stimulation was active or sham [22].

### 2.4. Measurements

Demographic data, including age, gender, body mass index (BMI; kg/m^2^), race, education, and marital status, as well as clinical data, including the index knee (the knee most affected), Kellgren–Lawrence score, and the average duration of OA (months), were collected. The measures utilized to assess clinical pain intensity, pain interference, and pain catastrophizing are described below. Pain outcomes were evaluated over 3 weeks, with assessments at the end of each week after five sessions.

NRS. The pain NRS is a subjective report on daily pain experiences, reflecting both the somatosensory and emotional dimensions of pain. The NRS was used by the participants to indicate a number between 0 (*no pain*) and 100 (*the worst pain imaginable*) to quantify their average pain over the past 24 h. The NRS is a reliable and well-validated measure known for its ability to accurately detect changes in pain among adults with knee OA [23].

The Western Ontario and McMaster Universities Osteoarthritis (WOMAC) Index pain subscale. The WOMAC Index is a validated tool utilized for assessing symptoms of knee OA, comprising 24 items categorized into three subscales: pain (5 items), stiffness (2 items), and functional disability (17 items) [24]. Each subscale demonstrates reliability and validity in evaluating knee OA patients [25–27]. Along with the NRS, average knee pain for the past 48 h was measured by the pain subscale, which consisted of 5 items on a 0–4 Likert scale measuring the pain severity during walking, climbing stairs, sleeping, resting, and standing. The participants' responses to each pain question were summed up to derive an aggregated score for pain intensity.

The WOMAC functional subscale. The functional subscale (17 items) has questions that cover everyday activities such as standing, walking, and using stairs. In this study, each participant's responses to these functional disability questions, rated on a 5-point scale (0 being none to 4 being extreme), were aggregated to produce a composite score for pain interference.

Pain Catastrophizing Scale (PCS). The PCS assesses participants' frequency of experiencing specific catastrophic thoughts or feelings when in pain across three dimensions: rumination (4 items), magnification (3 items), and helplessness (6 items) [28]. In this study, we used the PCS to obtain a state-based assessment to tap catastrophic cognitive processes during a pain experience, rather than using a trait-based catastrophizing assessment. The participants were instructed to refer to a specific current pain experience (i.e., present pain) when responding to the items [29–32]. Each item is rated on a 5-point scale ranging from “not at all” (0) to “all the time” (4). The total score was obtained by summing the raw scores across all items. The PCS demonstrated adequate internal consistency, with subscale alphas ranging from 0.66 to 0.87 (*α* for all items = 0.87) [28], and its sensitivity to psychosocial interventions for chronic pain has been established [33, 34].

### 2.5. Statistical Analysis

Descriptive statistics were used to characterize the study participants. The chi-square or Fisher's exact test for categorical variables and the *t*-test for continuous variables were used to compare participant characteristics between the groups. Multigroup LTA was used to achieve our primary aim. All analyses were conducted using Mplus Version 8.8 (Muthén and Muthén, Los Angeles, CA, USA). Supporting Table 1 includes the M*plus* syntax.

#### 2.5.1. LTA

LTA constitutes a type of structural equation model that allows for the examination and description of changes in categorical latent variables (i.e., latent statuses) over time (Collins and Lanza, 2010). LTA produces three sets of parameters: (1) a matrix of item-response probabilities at each time point (denoted as “*ρ*” parameters) that captures the likelihood of participants in each latent class to provide different responses to each continuous variable (e.g., clinical pain intensity), conditional on latent status membership; (2) a vector of latent status prevalences at Time 1 (“*δ*” parameters) describing the time-specific proportion of participants expected to belong to the latent class at each time point; and (3) matrices of transition probabilities (“*τ*” parameters) representing the probability of membership in a status at timepoint *t* dependent upon membership in a latent status at timepoint *t* − 1. In particular, these transition probabilities demonstrate patterns of change among the latent statuses and demonstrate how a latent status at one time point is likely to transition to another latent status at a different time point; in the matrix, the row corresponds to latent status membership at time, *t*, and the column corresponds to latent status membership at another time, *t* + 1. Additional details about LTA and its mathematical model are provided in Supporting Table 2.

In multigroup LTA, latent status prevalences (*δ*′s) and transition (*τ*′s) probabilities can be expressed as a function of a grouping variable, thus facilitating between-group comparisons of the prevalence of the latent statuses and incidence of transitions over time [35]. In the context of clinical trials, this approach enables the statistical evaluation of an intervention's efficacy. Theoretically speaking, each set of the aforementioned parameters potentially varies across study groups. To ensure interpretability, each element of the ρ-parameter matrix at Time 1 should be constrained to equate to the corresponding element at subsequent times, such that status definition at each timepoint remains consistent over time, thereby imposing *measurement invariance* [36]. This helps stabilize estimation to improve status identification and interpretation [16]. Furthermore, this facilitates group comparisons of both latent status and transition probabilities (*γ* and *τ* parameters, respectively) [36].

#### 2.5.2. Statistical Analysis in the Current Study

Model selection is a foremost and critical step in LTA. The common procedure involves not only the comparison of LTA models with different number of latent statuses considering model fit, but also parsimony and interpretability to identify the most suitable solution and the number of latent statuses. First, we fitted successive, unconditional LTA models—by running the models without covariates—to select the optimal number of latent statuses (constructed based on combinations of the four pain measures: the NRS, the WOMAC pain and functional scale, and the PCS). Models from 2–4 latent statuses were tested. The best-fitting model was selected based on statistical fit indices, such as the Akaike Information Criterion (AIC), Bayesian Information Criterion (BIC), sample-size adjusted BIC (SABIC), log-likelihood (LL), and entropy [37]. When fit statistics yielded contradictory information, we checked for the model that revealed latent statuses that made sense and were clinically meaningful. After determining the best-fitting model, multigroup LTA was conducted to model the transition probabilities of latent statuses over time and compare these differences between the active and sham groups. Herein, regarding measurement invariance (same item-response probabilities), we assumed that no difference existed in the way latent statuses were constructed across the follow-ups. To account for longitudinal study dropouts, the LTA treated incomplete data as “missing at random.” Accordingly, the procedure mapped the model parameters to all available data and the analyses were conducted using all available data points, through the full information maximum likelihood estimation, accounting for indicator- and longitudinal-level misses [38].

In addition to the LTA, this study conducted (1) a comparison of changes in the NRS, the WOMAC pain and functional scores, and the PCS scores from baseline to 3 weeks between the active and sham tDCS groups using the Wilcoxon rank-sum test, and (2) a latent profile analysis [39] to classify baseline pain profiles and compare outcomes across these profiles to obtain further Supporting information. The results of these analyses are detailed in Supporting Tables 3 and 4.

## 3. Results

The mean age was 65.32 ± 8.34 years in the active tDCS group and 66.60 ± 8.36 years in the sham tDCS group, with similar proportions of females in both. BMI values were 32.67 ± 8.66 kg/m^2^ for the active group and 35.52 ± 8.23 kg/m^2^ for the sham group. Almost half of the participants were White (43.3% in the active group and 53.3% in the sham group), over half were married or partnered (63.3% in the active group and 53.3% in the sham group), and most had education beyond a 2-year college degree (58.3% in the active group and 51.7% in the sham group). More details on clinical characteristics can be found in Supporting Table 5. No significant baseline differences were noted between these groups (*p* > 0.05). Additionally, there were no significant differences in any of the three pain domain measures at baseline (*p* > 0.05). The intervention was well tolerated by our participants with no significant side effects.

Table 1 presents fit statistics for unconditional LTA models with 2 to 4 statuses. Entropy favored a 3-status model, while other statistics recommended a 4-status model. However, the 4-status model did not significantly improve over the 3-status model, despite slightly better fit statistics. Inspection of both models showed that the 4-status model added one small, marginally different status with a relatively small number of participants and was difficult to interpret (< 5.0%), whereas the 3-status model revealed substantially differentiated and meaningful patterns of pain profiles. Thus, the 3-status model was identified as the most appropriate for the present data.

**Figure 2:**
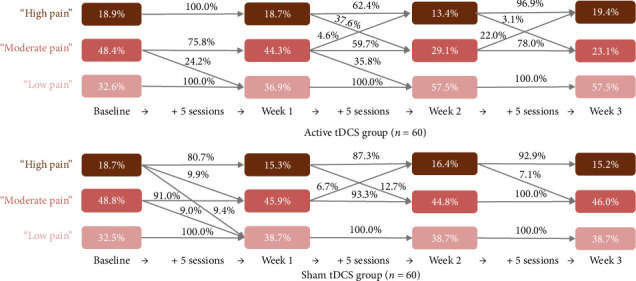
Transition probabilities and prevalence of latent statuses over time. *Note:* We labeled each status based on its item-response probabilities (referred to as “symptom indicator means” when the variable of interest is continuous as in our situation; i.e., the mean scores of the NRS, the WOMAC pain and functional scale, and the PCS); the first status was labeled “low pain,” the second was labeled “moderate pain,” and the third was labeled “high pain.”

Table 2 and Figure 2 present the multigroup LTA results. First, we labeled each status based on its item-response probabilities (referred to as “symptom indicator means” when the variable of interest is continuous as in our situation; i.e., the mean scores of the NRS, the WOMAC pain and functional scale, and the PCS); the first status was labeled “low pain,” the second was labeled “moderate pain,” and the third was labeled “high pain.” As the LTA model was specified to include measurement invariance over time, the definitions of the statuses remained consistent throughout the study. In both groups, the prevalence of “low pain” status generally increased over time, whereas that of both “moderate pain” and “high pain” generally decreased until the 3-week follow-up.

Table 3 and Figure 2 display group-specific transition probabilities across the active and sham groups. Participants in the active group who reported “moderate pain” status at baseline had a 24.2% probability of transitioning to “low pain” status after completing the Week 1 sessions. In contrast, participants in the sham group relatively remained stagnant during the same period.

Notable differences in transition probabilities between groups were observed from Week 1 to Week 2. After an additional five sessions by Week 2, active group participants who initially reported “high pain” status at Week 1 had a 37.6% probability of transitioning to “moderate pain” status. Similarly, those with “moderate pain” status at Week 1 had a 35.8% probability of transitioning to “low pain” status. In contrast, sham group participants had no likelihood of transitioning from “moderate pain” to “low pain” status during this period. Approximately 12.7% of participants in the sham group who reported “high pain” status at Week 1 transitioned to “moderate pain” status by Week 2. By Week 3, no notable transition dynamics between the groups were observed. However, active group participants who reported “moderate pain” status at Week 2 had a 22.0% likelihood of regressing to “high pain” status.

## 4. Discussion

Overall, active tDCS proved more effective at simultaneously improving all pain indicators in older adults than sham tDCS. Unlike previous research on targeted effects [10, 11], this holistic approach is critical as it addresses not only the physical sensation of pain but also the functional and psychological aspects that significantly impact the overall QoL in this demographic, ultimately enhancing our understanding of tDCS's “real-world” effectiveness. As these dimensions are known to be strongly correlated, improving all pain dimensions may also produce additive or synergistic effects. Moreover, our home-based modality renders continuous pain management more feasible and sustainable, supporting consistent treatment delivery to older adults with knee OA.

Notably, pain improvement was more evident by Week 2 (after 10 sessions), with no further noticeable effects observed by Week 3 (after five more sessions). This finding has significant research implications. Primarily, it underscores tDCS's potential to swiftly and effectively mitigate overall pain symptomatology, a crucial advantage in the current healthcare landscape where rapid solutions are imperative. Furthermore, the optimal effects of tDCS potentially depend on several factors, including stimulation parameters, electrode polarity, the target brain area, electrode preparation, and, importantly, treatment duration and intervals [40]. Our findings may prompt further discussion on the rationale behind the common practice of implementing up to 10 sessions [9, 40] and whether this frequency is sufficient to achieve optimal results. For example, an open-label study conducted by Ahn et al. [9] found that home-based, remotely supervised tDCS yielded a significant improvement in clinical pain (Cohen's d = 0.62, *p* < 0.01 for Visual Analog Scale pain; Cohen's d = 0.54, *p*=0.01 for WOMAC pain) following a 2-week treatment (10 daily home-based administration of 2-mA tDCS for 20 min) among 20 older adults with knee OA (mean age = 61.20 ± 7.23 years). Similarly, Ahn et al. [40] conducted a study involving 30 participants with symptomatic knee OA (mean age = 59.47 ± 6.91 years), who were randomly assigned to receive 10 daily home-based administration of 2-mA tDCS (20 min per session) paired with active mindfulness-based meditation (*n* = 15) or sham tDCS paired with sham mindfulness-based meditation (*n* = 15); the differences in clinical pain scores and OA-related symptom scores between the two groups were statistically significant (Cohen's d = 1.81, *p* < 0.000 for NRS; Cohen's d = 0.83, *p*=0.02 for WOMAC). This frequency of 10 daily home-based tDCS sessions has also been found to be effective in improving experimental pain sensitivity in older adults with knee OA [41, 42] and has been utilized for managing neuropsychiatric symptoms in older adults with Alzheimer's disease and related dementias [43]. Nonetheless, this interpretation requires caution, as a longer follow-up period (beyond 3 weeks) might have revealed substantial changes. Unfortunately, our team did not collect long-term follow-up data on pain catastrophizing.

Interestingly, the sham tDCS group also somewhat benefited; both groups exhibited positive transitions from “high pain” to “moderate pain.” These results suggest that a sham protocol previously considered inactive may also exert some minor neuromodulatory effects on certain outcomes [44], aligning with a few prior studies [45, 46]. Such a finding can be explained by skin sensations intentionally produced in the sham arm (ramp up/down) or cortical modulation by the microampere-scale current [45]. Yet most of the multigroup LTA findings were relatively descriptive; each status's transition probabilities did not have any verified statistical significance associated with them. Hence, our interpretations must be taken with caution.

Interestingly, a small portion of active tDCS participants belonging to the “low pain” status at Week 2 regressed to “high pain” status by Week 3. This finding underscores the need to intensively monitor and provide timely support to those at higher risk of regression, even during short-term follow-up. Unfortunately, LTA does not statistically identify individuals exhibiting specific transition patterns. Future investigations should focus on elucidating these individuals' profiles or characteristics, such as initial pain intensity, interference, and catastrophizing levels, which potentially influence tDCS responsiveness or the duration of its effects. Identifying these participant subgroups may enhance the design of sustainable tDCS interventions for older adults with knee OA in the long term.

### 4.1. Future Steps Based on the Current Study

Important future directions can be drawn from the current finding that relatively few active tDCS participants transitioned from “high pain” to “moderate pain” or “low pain” status compared with those transitioning from “moderate pain” to “low pain” status. Notably, among those belonging to the “high pain” status, the mean pain catastrophizing score was approximately 35, which exceeds the clinically relevant level of 30 for patients with chronic pain [47], while the scores for the other two groups were below 12. This finding may not be surprising as, especially, older adults with knee OA experience severe limitations in mobility and may be wheelchair-bound, which restricts their social activities. This limitation can lead to isolation and emotional distress, contributing to high pain catastrophizing [48]. Although more novel experimental designs are required to comprehensively explore the factors influencing intervention efficacy, this pain-related distress may affect the analgesic effects of tDCS. Indeed, catastrophizing thoughts can amplify pain perception and reduce the individual's ability to cope with pain, making it difficult for interventions to achieve their full potential. Therefore, an optimal intervention strategy for knee OA pain may, in the future, combine tDCS with other active interventions targeting psychological stress. Indeed, optimal treatments must incorporate a multidisciplinary approach for treating knee OA pain considering the concurrence of multiple clinical manifestations [4]. Several studies suggest combining tDCS with mindfulness meditation targeting attention, affect and stress [40], cognitive behavioral therapy [49], and/or exercise [50, 51].

### 4.2. Limitations and Research Implications

Our study has important limitations. First, as mentioned, the study followed all three pain domain measures in the short term. Future studies should extend the follow-up time to evaluate the longer term effects of tDCS on the overall pain experience, which is crucial for assessing its sustained benefits, cost-effectiveness, and time efficiency. Second, although our findings were quite noteworthy, future research should aim to increase the reliability of LTA by utilizing larger samples. This is necessary because, although there is no guideline for a minimum size of the general sample, a sufficient sample size can help generate a better estimate of LTA model parameters if the models have (a) homogenous classes, (b) high between-class separation, (c) large transition probabilities, (d) large class sizes at *t* = 1, and (e) a large overall sample size [52]. Smaller sample sizes may limit the stability across all time points, potentially causing model identification issues in the cross-sectional component of LTA and affecting the statistical power of the analysis [53]. Third, a critical oversight is that we did not comprehensively determine which participants, based on their characteristics, exhibited greater changes in pain or responsiveness to tDCS. This can be achieved by allowing covariates (time-invariant or time-varying covariates) to interact on the latent status (*δ*′s) and transition (*τ*′s) probabilities using an internal model-based approach, that is, LTA with covariates [54]. For instance, by employing this approach, researchers can ascertain how participants' race/ethnicity or socioeconomic status (i.e., time-invariant covariates) and physical or mental health status (i.e., time-varying covariates) are differentially associated with membership in change stages and transition probabilities during the intervention. This approach will be paramount to further tailoring intervention strategies. However, again, it was not going to be feasible in the present study as it necessitates a relatively large sample size. In cases where the sample size is small, some transitions between statuses may be less frequent from one time point to another, and certain statistics (e.g., odds ratios) cannot be accurately estimated [54]. Fourth, while our three pain domain measures provided valuable insights into pain experiences, future studies should include additional measures, such as anxiety and depression, to achieve a more comprehensive assessment of the overall pain experience of older adults with knee OA. Finally, we acknowledge that the term “pain catastrophizing” can be considered pejorative, stigmatizing, and conflicting with patient-centered care approaches [55]. Labeling patients in this manner can lead to blaming and stereotyping, adversely affecting decision-making and care quality. Recent analyses have proposed that terms such as “pain-related worrying” and “pain-related distress” can convey the intended meaning better than the term, “pain catastrophizing” [56].

## 5. Conclusion

The ability of multigroup LTA to simultaneously evaluate multiple pain domains within a single model and explore their changes as a function of intervention exposure facilitated a comprehensive assessment of tDCS's effects on pain in older adults with knee OA. Despite its limitations, we hope this study will serve as a basis for active discussions on the importance of addressing the overall pain experience of older adults with knee OA, thereby truly reflecting “real-world” pain management.

## Figures and Tables

**Table 1:** Fit statistics for unconditional LTA models.

No. of statuses	AIC	BIC	SABIC	LL	Entropy
2	11,510.289	11,579.977	11,500.938	−5730.145	0.923
3	11,198.384	11,312.671	11,183.049	−5558.192	**0.940**
4	**11,074.003**	**11,249.615**	**11,050.439**	**−5474.001**	0.920

*Note:* Optimal values in each column are written in bold font.

Abbreviations: AIC, Akaike Information Criteria; BIC, Bayesian Information Criteria; LL, log-likelihood; SABIC, sample-size adjusted BIC.

**Table 2:** The results of the unconditional LTA model with 3 latent statuses.

	Latent status		
	“Low pain”	“Moderate pain”	“High pain”
Symptom indicator means^a^			
Pain intensity (NRS)	28.23 ± 2.77	53.53 ± 2.37	67.64 ± 2.43
Knee pain intensity (WOMAC pain subscale)	4.25 ± 0.30	9.04 ± 0.34	11.60 ± 0.77
Knee pain interference (WOMAC functional subscale)	15.24 ± 1.29	31.53 ± 1.25	39.76 ± 2.54
Pain catastrophizing (PCS)	3.26 ± 0.59	11.68 ± 1.48	35.86 ± 1.92
Latent status membership prevalence			
Active tDCS			
Time 1 (baseline)	0.326	0.484	0.189
Time 2 (Week 1)	0.369	0.443	0.187
Time 3 (Week 2)	0.575	0.291	0.134
Time 4 (Week 3)	0.575	0.231	0.194
Sham tDCS			
Time 1 (baseline)	0.325	0.488	0.187
Time 2 (Week 1)	0.387	0.459	0.153
Time 3 (Week 2)	0.387	0.448	0.164
Time 4 (Week 3)	0.387	0.460	0.152

Abbreviations: NRS, Numeric Rating Scale; PCS, Pain Catastrophizing Scale; WOMAC, Western Ontario and McMaster Universities Osteoarthritis.

^a^Symptom indicator means constrained to be equal at baseline and at 1-week, 2-week, and 3-week follow-ups.

**Table 3:** Group-specific transition probabilities across the study groups.

	Latent status		
	“Low pain”	“Moderate pain”	“High pain”
Transition probabilities^a^ (rows for baseline, columns for Week 1)			
Active tDCS			
“Low pain”	**1.000**	0.000	0.000
“Moderate pain”	0.242	**0.758**	0.000
“High pain”	0.000	0.000	**1.000**
Sham tDCS			
“Low pain”	**1.000**	0.000	0.000
“Moderate pain”	0.090	**0.910**	0.00
“High pain”	0.094	0.099	**0.807**
Transition probabilities^a^ (rows for Week 1, columns for Week 2)			
Active tDCS			
“Low pain”	**1.000**	0.000	0.000
“Moderate pain”	0.358	**0.597**	0.046
“High pain”	0.000	0.376	**0.624**
Sham tDCS			
“Low pain”	**1.000**	0.000	0.000
“Moderate pain”	0.000	**0.933**	0.067
“High pain”	0.000	0.127	**0.873**
Transition probabilities^a^ (rows for Week 2, columns for Week 3)			
Active tDCS			
“Low pain”	**1.000**	0.000	0.000
“Moderate pain”	0.000	**0.780**	0.220
“High pain”	0.000	0.031	**0.969**
Sham tDCS			
“Low pain”	**1.000**	0.000	0.000
“Moderate pain”	0.000	**1.000**	0.000
“High pain”	0.000	0.071	**0.929**

Abbreviation. tDCS, transcranial direct current stimulation.

^a^Transition probabilities in bold font correspond to membership in the same latent status at both times.

## Data Availability

To guarantee the confidentiality and anonymity of the participants, data cannot be made publicly available. However, the data are available from the authors upon reasonable request. For more information, the corresponding author can be contacted.

## Supporting Information

The Supporting file includes the M*plus* syntax (statistical coding used in the current study), preliminary results showing changes in the NRS, WOAMC pain and functional scores, and PCS scores over time, as well as the detailed baseline characteristics of the participants.
