# Supplementary material for: Home-Based, Remotely Supervised Transcranial Direct Current Stimulation Improves the Overall Pain Experience of Older Adults With Knee Osteoarthritis
Source: Pain Res Manag. 2025 Feb 24;2025:1783171. doi: 10.1155/prm/1783171 (PMC11876529; doi:10.1155/prm/1783171)
Supplement: Supporting Information — Additional supporting information can be found online in the Supporting Information section. [file 1783171.f1.docx]

# **Supporting Information**

## **Supporting Table 1.** M*plus* syntax.

| Data:  FILE IS "C:\Users\XXXX\ XXXX\LTAmanuscript.csv";    Variable:  names are id nrs1 nrs2 nrs3 nrs4  womacp1 womacp2 womacp3 womacp4  womacpi1 womacpi2 womacpi3 womacpi4  pcs1 pcs2 pcs3 pcs4  treatment;  usevariables are white  nrs1 womacp1 womacpi1 pcs1  nrs2 womacp2 womacpi2 pcs2  nrs3 womacp3 womacpi3 pcs3  nrs4 womacp4 womacpi4 pcs4;  idvariable is id;  missing is .;  classes=w(2) c1(3) c2(3) c3(3) c4(3);  knownclass=w(treatment=0 treatment=1);  Analysis:  TYPE = MIXTURE;  STARTS = 500 200;  PROCESSORS = 2;  Model: %overall%  c2 c1 on w;  c3 c2 on w;  c4 c3 on w;  model w:  %w#1%  c2 on c1;  c3 on c2;  c4 on c3;  %w#2%  c2 on c1;  c3 on c2;  c4 on c3;  model c1:  %c1#1%  [nrs1](1);  [womacp1](2);  [womacpi1](3);  [pcs1](4);  %c1#2%  [nrs1](5);  [womacp1](6);  [womacpi1](7);  [pcs1](8);  %c1#3%  [nrs1](9);  [womacp1](10);  [womacpi1](11);  [pcs1](12);  model c2:  %c2#1%  [nrs2](1);  [womacp2](2);  [womacpi2](3);  [pcs2](4);  %c2#2%  [nrs2](5);  [womacp2](6);  [womacpi2](7);  [pcs2](8);  %c2#3%  [nrs2](9);  [womacp2](10);  [womacpi2](11);  [pcs2](12);  model c3:  %c3#1%  [nrs3](1);  [womacp3](2);  [womacpi3](3);  [pcs3](4);  %c3#2%  [nrs3](5);  [womacp3](6);  [womacpi3](7);  [pcs3](8);  %c3#3%  [nrs3](9);  [womacp3](10);  [womacpi3](11);  [pcs3](12);  model c4:  %c4#1%  [nrs4](1);  [womacp4](2);  [womacpi4](3);  [pcs4](4);  %c4#2%  [nrs4](5);  [womacp4](6);  [womacpi4](7);  [pcs4](8);  %c4#3%  [nrs4](9);  [womacp4](10);  [womacpi4](11);  [pcs4](12);  Output: tech15; |
| --- |

## **Supporting Table 2.** Mathematical model for latent transition analysis.

| Let L represent the categorical latent variable overall. L has S latent statuses.  Let L1 represent the categorical latent variable at Time 1, where s1 = 1, … S; let L2 represent the categorical variable at Time 2, where s2 = 1, … S; let L3 represent the categorical variable at Time 3, where s3 = 1, … S; let L3 represent the categorical variable at Time 4, where s4 = 1, … S. There are three parameters estimated in LTA:   1. **Item-response probabilities**, where *P* j, r _j,t_\|s_t_ represents the probability of response r _j, t_ to observe variable *j*, conditional on membership latent status s_t_ at Time *t*. For each combination of latent status s, observed variable *j*, and Time *t*, there are R_j_ item-response probabilities. Since each participant provides only one response, the probabilities of each of the response alternative to variable *j* sum to 1,   *Rj*  ∑ *P* j, r _j,t_\|s_t_ = 1  R *j ,t* = 1  The above calculation applies only when items have categorical responses. In the current study, however, the items are continuous variables (e.g., NRS, WOMAC pain subscale), so we have replaced ‘item-response probabilities’ with ‘symptom indicator means.’ Refer to **Table 2**.   1. **Latent status prevalence**, where δ_st_ represents the prevalence of latent status *s* at Time *t*. Latent statuses are mutually exclusive and exhaustive at each time point, hence each participant is a member of only one latent status at Time *t*,   *S*  ∑ δ_st_ = 1.  St = 1  For example, refer to **Table 2**. The sum of the prevalence of all latent statuses (“low pain,” “moderate pain,” and “high pain”) at a specific time *t* (e.g., time 3) equals 1 for the active tDCS group (0.575 + 0.291 + 0.134 = 1).   1. **Transition probabilities**, where T_st_ _+ 1_\|_St_ represent the probabilities of a transition to latent status s at Time T + 1, conditional on membership in latent status at Time *t*.   For example, refer to **Table 2**. For the sham tDCS group, T3_2_ \| 2_1_ = 0.099 represents the probability of transitioning to Latent Status 3 (i.e., “high pain”) at Time 2 (i.e., week 1), conditional on membership of Latent Status 2 (i.e., “moderate pain”) at Time 1 (i.e., baseline). |
| --- |

## **Supporting Table 3.** A comparison of the changes in the four pain measures between the two groups at 1 week, 2 weeks, and 3 weeks from the baseline.

| Variable | mean ± standard deviation | | Effect size (d) | Wilcoxon  statistic | *p*-value |
| --- | --- | --- | --- | --- | --- |
|  | Active tDCS  (n = 60) | Sham tDCS  (n = 60) |  |  |  |
| NRS change (week 1) | -9.12 ± 19.76 | -5.87 ± 18.12 | 0.17 | 1909.0 | 0.567 |
| NRS change (week 2) | -17.07 ± 25.08 | -7.43 ± 15.56 | 0.46 | 2186.5 | **0.042** |
| NRS change (week 3) | -24.07 ± 21.55 | -1.08 ± 16.46 | 1.20 | 2879.5 | **<0.001** |
| WOMAC pain subscale change (week 1) | -0.97 ± 2.83 | -1.13 ± 2.85 | -0.06 | 1746.5 | 0.779 |
| WOMAC pain subscale change (week 2) | -2.55 ± 2.88 | -1.58 ± 3.04 | 0.32 | 2175.0 | **0.047** |
| WOMAC pain subscale change (week 3) | -2.5 ± 3.15 | -1.98 ± 2.94 | 0.17 | 1929.0 | 0.497 |
| WOMAC functional subscale change (week 1) | -3.33 ± 6.83 | -3.20 ± 7.04 | 0.23 | 1787 | 0.948 |
| WOMAC functional subscale change (week 2) | -7.88 ± 8.40 | -5.25 ± 8.29 | 0.31 | 2081.5 | 0.140 |
| WOMAC functional subscale change (week 3) | -7.32 ± 10.48 | -5.21 ± 7.18 | 0.23 | 2059.0 | 0.174 |
| PCS change (week 1) | -3.90 ± 5.48 | -2.21 ± -2.15 | 0.25 | 2059.0 | 0.174 |
| PCS change (week 2) | -5.80 ± 8.91 | -2.87 ± -2.85 | 0.33 | 2195.0 | **0.038** |
| PCS change (week 3) | -4.43 ± 8.94 | -3.47 ± -3.47 | 0.11 | 1976.5 | 0.355 |

*Abbreviation.* NRS = numeric rating scale; PCS = pain catastrophizing scale; tDCS = transcranial direct current stimulation; WOMAC: Western Ontario and McMaster Universities Osteoarthritis

Significant results are indicated in **bold**.

**Supporting Table 4** presents a comparison of the changes in the four pain measures between the two groups at 1 week, 2 weeks, and 3 weeks from the baseline. The Wilcoxon rank-sum test revealed that the NRS changes from baseline to 2 weeks were significantly different between the active and sham tDCS groups (Cohen's d = 0.46; *p* = 0.042). The average decrease in NRS from baseline to 2 weeks was 17.07 ± 25.08 for the active group, while it was only 7.43 ± 15.56 for the sham group. Additionally, the NRS changes from baseline to 3 weeks were significantly different between the active and sham tDCS groups (Cohen's d = 1.20; *p* ≤ 0.001). The average decrease in NRS from baseline to 3 weeks was 24.07 ± 21.55 for the active group, compared to 1.08 ± 16.46 for the sham group. The changes in the WOMAC pain subscale from baseline to 2 weeks were also significantly different between the active and sham tDCS groups (Cohen's d = 0.32; *p* = 0.047). The average decrease in WOMAC pain from baseline to 2 weeks was 2.55 ± 2.88 for the active group and 1.58 ± 3.04 for the sham group. Finally, the changes in PCS from baseline to 2 weeks were significantly different between the active and sham tDCS groups (Cohen's d = 0.33; *p* = 0.038). The PCS average score decreased by 5.80 ± 8.91 at 3 weeks from baseline for the active group, compared to 2.87 ± 2.85 for the sham group. Although no significant changes in WOMAC functional subscale scores were observed between the two groups at any time point, the most notable changes occurred from baseline to 2 weeks: the average score decreased by 7.88 ± 8.40 at 2 weeks from baseline for the active group, compared to 5.25 ± 8.29 for the sham group.

## **Supporting Table 4-1.** Descriptive statistics of each group from baseline to week 3.

|  | mean ± standard deviation | | | | | |
| --- | --- | --- | --- | --- | --- | --- |
|  | Active tDCS (n = 60) | | | Sham tDCS (n = 60) | | |
|  | “Low pain”  (n = 13) | “Moderate pain”  (n = 28) | “High pain”  (n = 19) | “Low pain”  (n = 13) | “Moderate pain”  (n = 23) | “High pain”  (n = 24) |
| **NRS** | | | | | | |
| Time 1 (baseline) | 31.92 ± 14.48 | 52.14 ±15.95 | 75.16 ±14.02 | 29.46 ±16.93 | 45.65 ±17.09 | 66.88 ±14.13 |
| Time 2 (week 1) | 27.62 ± 17.90 | 42.39 ± 22.36 | 63.68 ± 20.32 | 22.38 ± 15.03 | 40.09 ± 19.65 | 61.38 ±20.31 |
| Time 3 (week 2) | 26.15 ± 17.44 | 37.36 ± 21.82 | 47.00 ± 23.08 | 21.69 ± 12.27 | 40.87 ± 21.95 | 57.08 ±17.26 |
| Time 4 (week 3) | 12.46 ± 9.25 | 29.54 ± 19.41 | 45.79 ± 21.46 | 25.00 ± 16.78 | 44.91 ± 24.75 | 67.29 ± 15.07 |
| **WOMAC pain subscale** | | | | | | |
| Time 1 (baseline) | 3.85 ± 1.46 | 8.54 ± 1.38 | 12.26 ±1.89 | 4.15 ± 1.35 | 8.39 ± 1.58 | 12.00 ±1.12 |
| Time 2 (week 1) | 4.23 ± 2.91 | 7.14 ±2.40 | 11.00 ± 2.96 | 4.62 ± 2.27 | 6.48 ± 1.93 | 10.75 ± 3.69 |
| Time 3 (week 2) | 2.46 ± 1.50 | 6.18 ± 2.61 | 8.63 ± 4.04 | 3.85 ± 2.25 | 6.13 ± 2.54 | 10.38 ± 4.07 |
| Time 4 (week 3) | 2.08 ± 1.59 | 5.89 ± 2.91 | 9.47 ± 4.49 | 3.31 ± 2.20 | 5.91 ± 2.62 | 9.88 ± 3.41 |
| **WOMAC functional subscale** | | | | | | |
| Time 1 (baseline) | 13.00 ± 6.96 | 27.43 ± 6.85 | 43.16 ± 5.15 | 17.46 ± 7.34 | 27.04 ± 6.77 | 41.33 ± 6.20 |
| Time 2 (week 1) | 11.69 ± 6.65 | 24.18 ± 6.58 | 38.32 ± 10.22 | 17.92 ±9.74 | 23.57 ± 8.41 | 36.42 ± 11.17 |
| Time 3 (week 2) | 8.77 ± 6.18 | 20.54 ± 7.13 | 31.32 ±11.60 | 13.85 ±6.84 | 21.52 ± 9.21 | 35.46 ± 11.25 |
| Time 4 (week 3) | 6.62 ± 5.86 | 19.07 ± 9.18 | 36.74 ±14.77 | 12.15 ± 7.41 | 22.96 ± 9.13 | 35.08 ± 9.53 |
| **PCS** | | | | | | |
| Time 1 (baseline) | 5.85 ± 3.63 | 11.32 ±9.23 | 28.74 ± 14.45 | 5.31 ± 4.87 | 12.87 ± 9.42 | 21.33 ± 12.88 |
| Time 2 (week 1) | 2.77 ± 3.02 | 7.25 ± 7.50 | 24.53 ± 15.34 | 4.38 ± 5.98 | 10.26 ± 8.31 | 18.79 ± 15.72 |
| Time 3 (week 2) | 2.31 ± 3.43 | 7.04 ± 8.96 | 19.16 ± 14.56 | 2.69 ± 3.31 | 9.91 ± 9.18 | 18.42 ± 13.70 |
| Time 4 (week 3) | 1.46 ± 2.93 | 7.07 ± 9.55 | 24.00 ± 15.94 | 3.31 ± 4.06 | 8.87 ± 8.68 | 17.58 ± 13.91 |

Latent profile analysis was conducted using M*plus* version 8.8. This analysis determined the number and nature of latent profiles of pain based on patient responses to four measures (NRS, WOMAC pain and functional subscale, and PCS). Selection of the optimal number of profiles relied on several statistical fit indices, such as the Akaike Information Criterion (AIC), Bayesian Information Criterion (BIC), Sample-size Adjusted BIC (SABIC), Log-Likelihood (LL), and entropy. The 3-profile model yielded stable profiles (AIC = 3581.02, BIC = 3667.43, SABIC = 3569.42, LL = -1759.51, entropy = 0.997). Based on the results, profile 1 was labeled as “low pain,” profile 2 as “moderate pain,” and profile 3 as “high pain.” **Supporting Table 4-1** shows the descriptive statistics of each group from baseline to week 3.

## **Supporting Table 4-2.** A comparison of the changes in the four pain measures across the three latent profiles at 1 week, 2 weeks, and 3 weeks from baseline.

|  | “Low pain” | | | | | “Moderate pain” | | | | | “High pain” | | | | |
| --- | --- | --- | --- | --- | --- | --- | --- | --- | --- | --- | --- | --- | --- | --- | --- |
| Variable | mean ± standard deviation | | Effect size (d) | Wilcoxon  statistic | *p*-value | mean ± standard deviation | | Effect size (d) | Wilcoxon  statistic | *p*-value | mean ± standard deviation | | Effect size (d) | Wilcoxon  statistic | *p*-value |
|  | Active tDCS  (n = 13) | Sham tDCS  (n = 13) |  |  |  | Active tDCS  (n = 28) | Sham tDCS  (n = 23) |  |  |  | Active tDCS  (n = 19) | Sham tDCS  (n = 24) |  |  |  |
| NRS change (week 1) | -4.31 ± 13.3 | -7.08 ± 11.4 | -0.22 | 68 | 0.407 | -9.75 ± 21.4 | -5.57 ± 23.2 | 0.19 | 339.5 | 0.747 | -11.50 ± 21.7 | -5.50 ± 16.5 | 0.32 | 288.5 | 0.140 |
| NRS change (week 2) | -7.77 ± 11.1 | -5.77 ± 17.5 | -0.14 | 80 | 0.837 | -14.8 ± 23.1 | -4.78 ± 20.4 | 0.46 | 389.5 | 0.203 | -28.20 ± 29.4 | -9.79 ± 12.5 | 0.85 | 329.5 | **0.012** |
| NRS change (week 3) | -19.5 ± 16.3 | -4.46 ± 19.5 | 0.98 | 126.5 | **0.033** | -22.6 ± 22.1 | -0.74 ± 21.2 | 1.00 | 485.5 | **0.002** | -24.40 ± 23.8 | 0.42 ± 12.2 | 1.64 | 407.5 | **<0.001** |
| WOMAC pain subscale change (week 1) | 0.39 ± 3.01 | 0.46 ± 1.76 | 0.03 | 89 | 0.836 | -1.39 ± 2.39 | -1.91 ± 2.19 | -0.23 | 273 | 0.354 | -1.26 ± 3.21 | -1.25 ± 3.59 | 0.003 | 227 | 0.990 |
| WOMAC pain subscale change (week 2) | -1.38 ± 1.45 | -0.31 ± 2.29 | 0.56 | 108.5 | 0.218 | -2.36 ± 2.56 | -2.26 ± 2.43 | 0.04 | 347.5 | 0.632 | -3.63 ± 3.76 | -1.62 ± 3.79 | 0.53 | 300.5 | 0.077 |
| WOMAC pain subscale change (week 3) | -1.77 ± 1.96 | -0.85 ± 2.51 | 0.41 | 100.5 | 0.421 | -2.64 ± 2.79 | -2.48 ± 2.45 | 0.06 | 336 | 0.796 | -2.79 ± 4.28 | -2.12 ± 3.53 | 0.17 | 241.5 | 0.749 |
| WOMAC functional subscale change (week 1) | -1.31 ± 5.33 | 0.46 ± 4.45 | 0.36 | 95.5 | 0.589 | -3.25 ± 6.52 | -3.48 ± 4.67 | -0.04 | 313 | 0.872 | -4.84 ± 8.21 | -4.92 ± 9.34 | -0.01 | 222.5 | 0.902 |
| WOMAC functional subscale change (week 2) | -4.23 ± 6.47 | -3.62 ± 5.16 | 0.11 | 83.5 | 0.980 | -6.89 ± 6.83 | -5.52 ± 6.56 | 0..20 | 359 | 0.489 | -11.8 ± 10.5 | -5.88 ± 11.1 | 0.55 | 297.5 | 0.090 |
| WOMAC functional subscale change (week 3) | -6.38 ± 7.46 | -5.31 ± 7.91 | 0.14 | 92.5 | 0.700 | -8.36 ± 8.86 | -4.09 ± 5.99 | 0.55 | 431.5 | **0.038** | -6.42 ± 14.4 | -6.25 ± 8.06 | 0.02 | 223.5 | 0.922 |
| PCS change (week 1) | -3.08 ± 3.38 | -0.92 ± 6.08 | 0.44 | 110 | 0.195 | -4.07 ± 5.00 | -2.61 ± 6.46 | 0.26 | 382.5 | 0.254 | -4.21 ± 7.36 | -2.54 ± 9.96 | 0.19 | 248 | 0.633 |
| PCS change (week 2) | -3.54 ± 3.57 | -2.62 ± 3.18 | 0.27 | 107 | 0.253 | -4.29 ± 8.81 | -2.96 ± 5.36 | 0.18 | 356 | 0.525 | -9.58 ± 10.8 | -2.92 ± 12.5 | 0.56 | 313.5 | **0.037** |
| PCS change (week 3) | -4.38 ± 3.38 | -2.00 ± 4.06 | 0.64 | 130 | **0.020** | -4.25 ± 9.97 | -4.00 ± 6.36 | 0.03 | 338 | 0.768 | -4.74 ± 10.5 | -3.75 ± 12.8 | 0.08 | 227.5 | 1.000 |

*Abbreviation.* NRS = numeric rating scale; PCS = pain catastrophizing scale; tDCS = transcranial direct current stimulation; WOMAC: Western Ontario and McMaster Universities Osteoarthritis

Significant results are indicated in **bold**.

**Supporting Table 4-2** presents a comparison of the changes in the four pain measures across the three latent profiles at 1 week, 2 weeks, and 3 weeks from baseline. The Wilcoxon rank-sum test revealed that there were significant changes in the NRS from baseline to 3 weeks between the active and sham tDCS groups across all profiles (*p* < .050). In the “low pain” profile, PCS changes from baseline to 3 weeks were significantly different between the active and sham tDCS groups (Cohen's d = 0.64; *p* = 0.020). In the “moderate pain” profile, WOMAC functional subscale changes from baseline to 3 weeks were significantly different between the active and sham tDCS groups (Cohen's d = 0.55; *p* = 0.038). For the “high pain” profile, the most notable changes occurred from baseline to 2 weeks. For example, NRS changes from baseline to 2 weeks were significantly different between the active and sham tDCS groups (Cohen's d = 0.85; *p* = 0.012). Although no significant changes were observed in WOMAC pain and functional scores, most reductions in pain measures were noted by week 2 based on Cohen’s d and Wilcoxon statistics. Additionally, PCS changes from baseline to 2 weeks were significantly different between the active and sham tDCS groups in the “high pain” profile (Cohen's d = 0.56; *p* = 0.037).

## **Supporting Table 5.** Basic characteristics of the participants (N = 120).

|  | n (%) or mean ± standard deviation | | *p*-value |
| --- | --- | --- | --- |
|  | Active tDCS (n = 60) | Sham tDCS (n = 60) |  |
| Age, years | 65.32 ± 8.34 | 66.60 ± 8.36 | 0.405 |
| Gender |  |  | 0.844 |
| male | 20 (33.3%) | 18 (30.0%) |  |
| female | 40 (66.7%) | 42 (70.0%) |  |
| Body mass index (kg/m^2^) | 32.67 ± 8.66 | 35.52 ± 8.23 | 0.924 |
| Race^a^ |  |  | 0.361 |
| white | 26 (43,3%) | 32 (53.3%) |  |
| non-white | 34 (56.7%) | 28 (46.7%) |  |
| Marital status |  |  | 0.354 |
| married/partnered | 38 (63.3%) | 32 (53.3%) |  |
| nonmarried/unpartnered | 22 (36.7%) | 28 (46.7%) |  |
| Education |  |  | 0.582 |
| two-year college degree or less | 25 (41.7%) | 29 (48.3%) |  |
| four-year college degree of higher | 35 (58.3%) | 31 (51.7%) |  |
| Index knee |  |  | 0.713 |
| right | 35 (58.3%) | 32 (53.3%) |  |
| left | 25 (41.7%) | 28 (46.7%) |  |
| Kellgren-Lawrence score |  |  | 0.371 |
| 1 | 9 (15.0%) | 7 (11.7%) |  |
| 2 | 20 (33.3%) | 20 (33.3%) |  |
| 3 | 22 (36.7%) | 29 (48.3%) |  |
| 4 | 9 (15.0%) | 4 (6.7%) |  |
| Average duration of osteoarthritis (months) | 71.35 ± 75.23 | 69.25 ± 82.18 | 0.885 |
| Pain intensity (NRS) | 55.05 ± 21.78 | 50.63 ± 21.59 | 0.271 |
| Knee pain intensity (WOMAC pain subscale) | 8.70 ± 3.41 | 8.92 ± 3.27 | 0.725 |
| Knee pain interference (WOMAC functional subscale) | 29.28 ± 11.53 | 30.68 ± 12.68 | 0.432 |
| Pain catastrophizing (PCS) | 15.65 ± 13.88 | 14.62 ± 11.98 | 0.666 |

^a^Fisher's exact test

*Abbreviation.* NRS = numeric rating scale; PCS = pain catastrophizing scale; tDCS = transcranial direct current stimulation; WOMAC: Western Ontario and McMaster Universities Osteoarthritis
